# Retrieving Binary Answers Using Whole-Brain Activity Pattern Classification

**DOI:** 10.3389/fnhum.2015.00689

**Published:** 2015-12-23

**Authors:** Norberto E. Nawa, Hiroshi Ando

**Affiliations:** ^1^Brain Networks and Communication Laboratory, Center for Information and Neural Networks, National Institute of Information and Communications TechnologyOsaka, Japan; ^2^Multisensory Cognition and Computation Laboratory, Universal Communication Research Institute, National Institute of Information and Communications TechnologyKyoto, Japan; ^3^Graduate School of Frontier Biosciences, Osaka UniversityOsaka, Japan

**Keywords:** machine learning classification, mental tasks, disorders of consciousness, MVPA, fMRI

## Abstract

Multivariate pattern analysis (MVPA) has been successfully employed to advance our understanding of where and how information regarding different mental states is represented in the human brain, bringing new insights into how these states come to fruition, and providing a promising complement to the mass-univariate approach. Here, we employed MVPA to classify whole-brain activity patterns occurring in single fMRI scans, in order to retrieve binary answers from experiment participants. Five healthy volunteers performed two types of mental task while in the MRI scanner: counting down numbers and recalling positive autobiographical events. Data from these runs were used to train individual machine learning based classifiers that predicted which mental task was being performed based on the voxel-based brain activity patterns. On a different day, the same volunteers reentered the scanner and listened to six statements (e.g., “the month you were born is an odd number”), and were told to countdown numbers if the statement was true (yes) or recall positive events otherwise (no). The previously trained classifiers were then used to assign labels (yes/no) to the scans collected during the 24-second response periods following each one of the statements. Mean classification accuracies at the single scan level were in the range of 73.6 to 80.8%, significantly above chance for all participants. When applying a majority vote on the scans within each response period, i.e., the most frequent label (yes/no) in the response period becomes the answer to the previous statement, 5.0 to 5.8 sentences, out of 6, were correctly classified in each one of the runs, on average. These results indicate that binary answers can be retrieved from whole-brain activity patterns, suggesting that MVPA provides an alternative way to establish basic communication with unresponsive patients when other techniques are not successful.

## Introduction

The mass-univariate approach has been employed in a substantial portion of functional magnetic resonance imaging (fMRI) studies, where several thousands of voxels are independently examined for changes in brain activity level as measured by blood oxygenation-level dependent (BOLD) signal, in order to identify the locations in the brain that are associated with different mental processes. In contrast, multivariate pattern analysis (MVPA) looks at the activity patterns formed across voxels that are located within a region or over the entire brain, and how these patterns can be used to predict the underlying psychological processes or mental states experienced by a subject. By heavily employing machine learning techniques, MVPA can simultaneously look at the contribution of multiple voxels, providing a complementary and often more sensitive alternative than univariate methods to understand where and how information regarding different mental states is represented in the brain (Jimura and Poldrack, 2012). Moreover, it offers the possibility of examining the association between brain activity and behavior on a trial-by-trial basis within much shorter time durations (Norman et al., 2006), as opposed to the mass-univariate approach where typically trials spanning over much longer periods of time are so to say averaged, in order to compute a statistic that will determine whether a given voxel has responded or not to the experimental manipulation.

Multivariate pattern analysis has been employed to, among other things, identify patterns of brain activity that attempt to predict the type of the viewed stimulus (Haxby et al., 2001) or whether subjects perceived face images as being familiar or novel (Rissman et al., 2010). Moreover, the multivariate approach has shown to be able to effectively discriminate between different mental states even in the absence of external stimuli, such as estimating the subject’s emotional state (Sitaram et al., 2011) or determining individual episodic memories (Chadwick et al., 2010). However, a much less explored field is using MVPA as a means to enable simple communication based on the brain activity of scanned subjects. Following the mass-univariate tradition, previous attempts to employ fMRI to communicate with healthy volunteers or patients have mostly relied on detecting brain activity modulations in specific regions of the brain over a few tens of seconds at a time. In a study by Monti et al. (2010), 54 patients with severe brain injury (23 in a vegetative state, and 31 in a minimally conscious state) underwent fMRI scanning runs where they were cued to alternate between 30 s of an imagery task and 30 s of rest. Two imagery tasks were employed; a motor imagery task, where participants were instructed to imagine to be standing still in a tennis court, while hitting a ball back and forth to an imagined instructor, and a spatial imagery task, where participants were told to imagine to be navigating the streets of a familiar city or the rooms in their home. Results of a mass-univariate general linear model analysis (GLM) contrasting the imagery periods with the rest periods in a group of 16 healthy controls showed that those tasks reliably elicited distinct fMRI responses in the supplementary motor area (motor imagery) and the parahippocampal gyrus (spatial imagery). Using the same tasks, five patients appeared to be able to modulate their brain activity in accordance with the cues. The experimenters further attempted to retrieve binary answers to questions such as “do you have brothers?” from 16 controls and 1 patient, by instructing them to perform one imagery task if the answer was ‘yes’ and the other one if the answer was ‘no’. Answers were inferred by using a similarity metric to assess how much the elicited brain activity patterns in two regions of interest during the response periods matched the patterns observed while participants simply alternated between the imagery tasks and rest. Answers retrieved from healthy controls were 100% accurate (three questions, chance level 50%, 5 min required to retrieve one answer). Six questions were asked to the patient, and to 5 of them the inferred answers matched the factual answers.

Sorger et al. (2012) developed a real-time spelling system where characters were encoded using a combination of three mental tasks, three onset delays (0, 10, 20 s), and three durations (10, 20, 30 s). Twenty-seven uniquely identifiable fMRI timecourse responses could be generated under that scheme, which were then used to encode 26 different letters plus the blank space. Results from six healthy participants indicated that the first letter choice as determined by an automated decoder was correct in 82% of the cases. If the second and third letter choices were also considered, the correct letter appeared in the subset of candidates in 95 and 100% of the time, respectively. Using the top three letter candidates output by the decoder plus the contextual information provided by the question, experimenters were able to decipher the correct answer 100% of the time (two questions, chance level 3.7% per character, 50 s required to retrieve one character).

Naci et al. (2013) proposed an alternative scheme inspired by the oddball paradigm to retrieve binary answers based on fMRI activity. Working on the premise that selective attention enhances brain responses to attended sounds, they first performed experiments to localize the brain regions where activity was intensified when participants paid attention to specific target words (“yes” or “no”) that appeared within a sequence of auditory stimuli interspersed with distractors (“1,” “2,” …, “9”). Each time a sequence was played, it would contain repetitions of only one of the target words. Data from the localizer sessions was used to identify the two most strongly activated regions, on an individual basis, when participants attended to the target words. During the communication sessions, participants would listen to a question followed by the two auditory stimuli sequences used in the localizer sessions. In order to convey the answer to a question, participants were told to attend to the occurrences of the word that corresponded to the factual answer (e.g., “yes”), while ignoring the occurrences of the opposite word (e.g., “no”). Questions were repeatedly asked during a session, and fMRI activity modulations within the previously identified regions were used to determine whether the answer to the question was ‘yes’ or ‘no’. Results from 15 healthy participants showed that the responses were correct 90% of the time (two questions, chance level 50%, approximately 5 min required to retrieve one answer).

Though several brain-based communication have been proposed to establish communication with patients in a vegetative state or minimally conscious state, it still remains to be verified whether binary answers can be retrieved from whole-brain activity patterns using MVPA. Here, instead of focusing on brain activity modulations in specific brain regions or networks, we employed the multivariate approach to classify whole-brain activity patterns on single fMRI scans. MVPA capitalizes on the fMRI’s capacity of collecting data from thousands of brain locations at the same time, by looking at brain activity patterns formed across voxels or regions. That naturally circumvents the need to make *a priori* assumptions about where in the brain distinctive modulations should be observed. Machine learning based classification of single whole-brain fMRI scans has shown to be capable of achieving fairly high levels of classification accuracy (Mourão-Miranda et al., 2005; Laconte et al., 2007; Nawa and Ando, 2014). Hence, we investigated whether that could be used for the acquisition of binary responses to simple questions, potentially paving the way for faster, and thus more natural and fluid, fMRI based communication systems with patients with disorders of consciousness.

## Materials and Methods

### Participants

This study was approved by the National Institute of Information and Communications Technology institutional review board and carried out in accordance with the Declaration of Helsinki. We recruited six healthy right-handed volunteers (21–24 years old, one female, all fluent in Japanese) by sending a call for participation to a mailing list formed by local college students potentially interested in taking part in research studies performed on campus. All volunteers signed informed consent before participation and were remunerated for their time. One male participant failed to perform the task properly so all analyses were performed on the data of the remaining five participants.

### Experimental Protocol and Task Instructions

Participants took part in the study over the course of 2 days, with on average 4 days in between. In the first day (Day 1), participants entered the MRI scanner and alternated between two mental tasks, the Countdown task and the Positive Autobiographical Memory (PAM) task (Nawa and Ando, 2014). For the Countdown task, participants were instructed to start mentally counting down from 100 as soon as the relevant auditory cue was heard (“1” in English, male speaker, duration of 398 ms). They were also told to perform the task at a comfortable pace, such as one subtraction every second, and not to worry about occasional mistakes. Participants were asked to keep counting until they heard the auditory cue signaling them to stop (pure tone, 440 Hz, duration 398 ms).

For the PAM task, participants were asked to prepare beforehand a list of about five positive personal events that they had experienced in their lives. To illustrate examples of positive events, we told participants to select occasions involving people, places or things that they liked and enjoyed, or which were associated with feelings of happiness, satisfaction, and elation. No restrictions were placed regarding the contents of their memories or the recency of the events on which the memories were based. On the day of the study, participants were asked to select events in the list to reminisce about during the experiment. They were told to start a constant flow of recollections as soon as the relevant auditory cue was heard (“2” in English, male speaker, duration of 398 ms), and maintain that flow until they were cued to stop (same pure tone). We suggested to the participants to avoid switching between different events too often, as we thought that would render the task unnecessarily difficult, though they were free to do that at their will.

**FIGURE 1:**
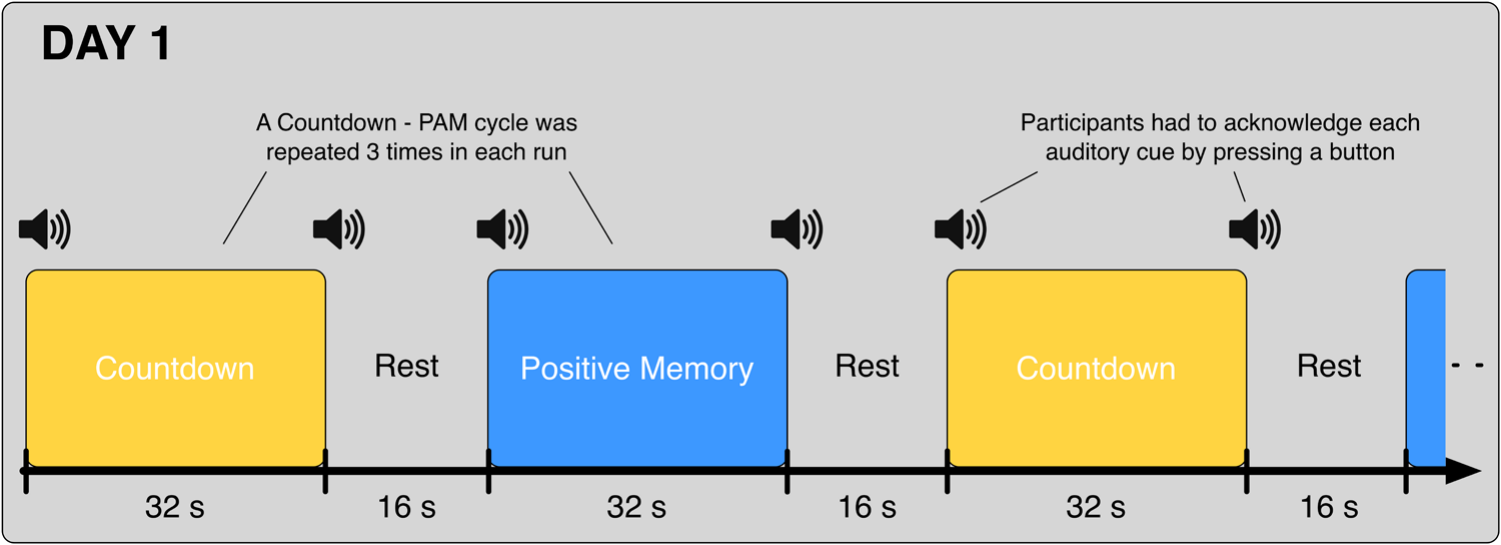
**Schematic of the alternating Countdown - Positive Autobiographical Memory (PAM) task.** Auditory cues were given at the start and end of each block. Participants were instructed to acknowledge each cue with a button press, and immediately start/stop the execution of the corresponding task. On Day 1, each participant underwent eight runs of the Countdown – PAM task. Data from these runs were used to train the classifiers.

On Day 1, participants underwent 8 fMRI runs. In each one of the runs, participants alternated between the Countdown task (32 s) and the PAM task (32 s), interleaved with 16-second rest periods in between; this cycle was repeated three times in each run (**Figure 1**). Participants were instructed to press the button and acknowledge each one of the auditory cues as fast and as accurately as possible; data from the blocks where the task-start auditory cues were not acknowledged were excluded from the analysis.

**FIGURE 2:**
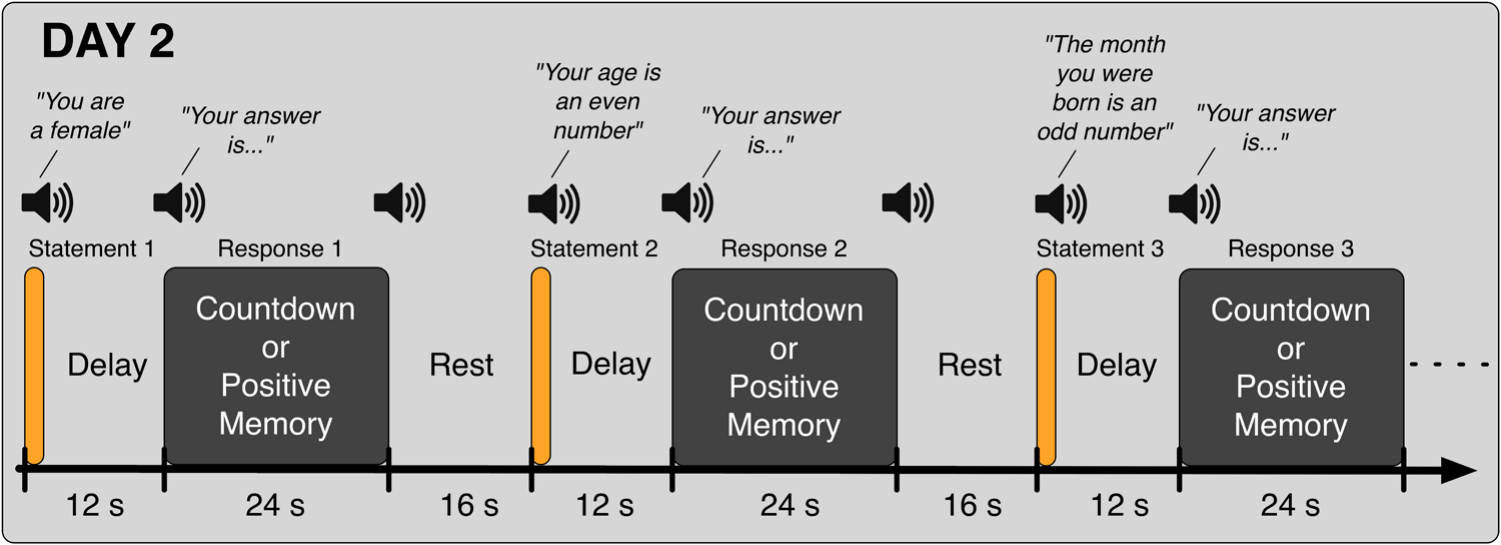
**Schematic of the Binary Answer task.** Six sentences were played on each run, and 12 s after the onset of each sentence, participants were prompted to provide an answer by executing the appropriate task, Countdown (yes) or PAM (no), for 24 s. The next sentence was played following a rest period of 16 s. Participants were instructed to acknowledge all auditory cues.

In the second day (Day 2), participants came back to the laboratory and again took part in eight fMRI runs. In the first five runs, they performed the Binary Answer task (**Figure 2**); participants listened to sentences such as “Your age is an even number” (in Japanese, female speaker, durations in the range of 1600 to 2680 ms), and 12 s after the onset of the sentence, they were prompted to provide an answer (“Your answer is…” in Japanese, female speaker, duration of 855 ms) by performing the Countdown task if the sentence was factually correct (yes) or the PAM task otherwise (no). Twenty four seconds later, an auditory cue (same pure tone) was delivered to signal them to stop performing the mental task. The next sentence was played after a 16-s rest period. Participants were instructed to respond to all auditory cues with a button press. Six different sentences were played in each run of the Binary Answer task (see **Table 1** for a list of all sentences). The order of the sentences was the same across all five runs of the Binary Answer task, and for all participants. In the last three runs on Day 2, participants performed the Countdown – PAM task identically as in Day 1. At debriefing time, we asked the participants to describe what they reminisced about during the PAM blocks in the Countdown-PAM task, and to rate the *pleasantness* and *vividness* of those memories using a numerical scale. In addition to that, on Day 2 we asked participants to describe and rate the memories used to signal a ‘no’ (PAM), and to provide factual answers to the six sentences played during the experiment (**Table 1**). The answers were used solely to assess the performance of the machine learning based classifiers, and were not used anywhere else in the analysis.

**Table 1:** Sentences used in the Binary Answer task on Day 2 (English translations).

		P1	P2	P3	P4	P5
S1	“You are a female”	Yes	No	No	No	No
S2	“Your age is an even number”	No	Yes	No	No	Yes
S3	“The month you were born is an odd number”	Yes	Yes	Yes	Yes	No
S4	“Your age is an odd number”	Yes	No	Yes	Yes	No
S5	“You are a male”	No	Yes	Yes	Yes	Yes
S6	“The month you were born is an even number”	No	No	No	No	Yes


*Columns P1-P5 show the factual answers provided by each participant at debriefing time.*

### Data Acquisition

Brain imaging data was acquired using a 3T Siemens Magnetom Trio, A Tim System scanner (Siemens Healthcare, Erlangen, Germany) equipped with a 32-channel standard head coil. Participants lay supine in the scanner and wore padded headphones, from where instructions and auditory cues were binaurally delivered. Behavioral responses were given via a MRI-compatible response pad (right index finger) connected to a computer that logged the button presses. The same computer ran a program written in Presentation (Neurobehavioral Systems, Inc., Albany, CA, USA) that controlled the delivery of the auditory cues in agreement with the acquisition of functional images. Right before the start of the experimental runs, T2-weighted anatomical images were acquired in the same plane as the functional images using a turbo spin echo sequence (TR = 6000 ms, TE = 57 ms, FA = 90°, FOV = 192 mm × 192 mm, matrix size = 256 × 256, in-plane resolution 0.75 mm × 0.75 mm). In each of the alternating Countdown – PAM task runs, 147 whole-brain echo-planar functional images were acquired in 30 contiguous 4 mm axial slices (1-mm gap) parallel to the AC-PC line (TR = 2000 ms, TE = 30 ms, FA = 80°, FOV = 192 mm × 192 mm, matrix size = 64 × 64, in-plane resolution 3 mm × 3 mm). In each one of the Binary Answer task runs, 159 whole-brain images were acquired using the same scanning parameters. Each run of the Countdown – PAM task and Binary Answer task were 294 and 318 s long, respectively. Before analyses, the first three scans of each session were discarded to account for magnetic saturation effects. Whole-brain T1-weighted anatomical images (1 mm^3^) were acquired after the last experimental run of either Day 1 or Day 2.

To minimize the effects of physiological noise in the imaging data, cardiac, and respiratory data were recorded during scanning (AD Instruments, Dunedin, New Zealand). Cardiac data was monitored using a piezoelectric pulse transducer attached to the index finger of the participant’s left hand, while respiration was monitored using a transducer belt strapped around the upper abdomen. The sampling rate of both signals was 1 kHz; the trigger signal output by the scanner at the start of each functional image acquisition was recorded to enable temporal registration of the cardio-respiratory data streams to the brain imaging data.

### Data Preprocessing

Cardiac and respiratory waveforms were visually inspected to confirm that there were no major problems with the measurements. In-house routines written in Matlab (version R2007a, Mathworks, Inc., Natick, MA, USA) were used to determine the cardiac trigger times from the waveforms. RETROICOR (Glover et al., 2000) was applied to the imaging data to reduce the effects of cardiac and respiratory cycles. In addition, clean-up techniques based on estimated respiratory (Birn et al., 2008) and cardiac (Chang et al., 2009) response functions were employed to regress low-frequency BOLD signal fluctuations due to variations in breathing and heart rates. The resulting functional scans were then preprocessed using SPM 5 (Wellcome Trust Centre for Neuroimaging, UK, http://www.fil.ion.ucl.ac.uk/spm/software/spm5): slice timing correction was performed using the first slice as a reference, followed by realignment and adjustment of head motion using the first scan of each run as a reference, after realigning the first scan of each run to the first scan of the first run; functional and anatomical images were co-registered using a two-step procedure involving the participant’s T2- and T1-weighted anatomical images. Functional images were spatially normalized to the standard stereotaxic Montreal Neurological Institute (MNI) space by applying the transformation matrix derived from the normalization of the T1-weighted anatomical image to the SPM 5 *templates/T1.nii* image. The original voxel size was kept the same throughout these steps (3 mm × 3 mm × 5 mm) resulting in images of size (53, 63, 28) voxels in the (X, Y, Z) dimensions, respectively, after spatial normalization. From here, two different processing pipelines were used to prepare the data for the general linear model (GLM) analysis and the MVPA. Scans used in the GLM analysis were spatially smoothed using a Gaussian kernel of 8-mm full width at half maximum (FWHM). For the data used in the MVPA, there were four additional steps after spatial normalization. First, nuisance variables were regressed from the BOLD time series of each voxel: the six affine head motion parameters estimated during the realignment step, the mean time series of a region corresponding to white matter (3-mm sphere centered at MNI coordinates *x* = 26, *y* = -12, *z* = 35), and cerebrospinal fluid (CSF) (3-mm sphere centered at MNI coordinates *x* = 19, *y* = -33, *z* = 18), the mean time series across the whole-brain (global signal), computed by using a binary mask generated by thresholding the SPM 5 image *apriori/grey.nii* at 0.22, plus a constant regressor for each one of the sessions to account for the mean session effect. Next, the BOLD time series of each voxel was high-pass filtered (cut-off frequency of 0.008 Hz), and the voxel values recorded within a session were scaled to a grand mean of 100. Finally, the BOLD time series of each voxel was standardized by subtracting the mean and dividing it by the standard deviation, with both values computed from the time series of the respective voxel over the entire experiment (day).

### General Linear Model Analysis

A GLM analysis was performed to identify voxels that were preferentially engaged by the Countdown task and the PAM task based on the data collected on Day 1. Results of this analysis were used to perform feature selection in the subsequent MVPA. At the individual-level, brain activity during the execution of the mental tasks was estimated for each voxel using the GLM implemented in SPM 5. The time series for each voxel was high-pass filtered to 0.0078 Hz, and serial correlations were corrected by an autoregressive AR(1) model.

The GLM had two regressors of interest corresponding to the two mental tasks performed in each run. Regressors of no interest were the six parameters describing head motion, derived during realignment, plus the constant regressors accounting for each individual run. The brain activity elicited during the execution of the mental tasks was modeled by a boxcar function of 32 s of duration, positioned at the onset times of the acknowledged blocks, convolved with the canonical hemodynamic response function provided in SPM 5. Linear contrast images were generated for each participant using pairwise comparisons between tasks. The resulting statistical maps were submitted to two voxel-level threshold to test for significance; *p* < 0.001, uncorrected (P001), and a more conservative criterion, *p* < 0.05, corrected for multiple comparisons using family wise error (FWE05). Two binary masks, one for each threshold level, were generated for each participant by taking the union of the surviving voxels yielded by the contrasts Countdown > PAM and PAM > Countdown. Because the spatial maps resulting from these contrasts were used to select voxels for the subsequent multivariate pattern analysis, masks were generated using data from the odd runs (1, 3, 5, and 7) and from the even runs (2, 4, 6, and 8), in order to separate the data used to select the voxels (features) from the data used in the main analysis (Kriegeskorte et al., 2009).

### Multivariate Pattern Analysis

The 116 spatial masks of the Automated Anatomical Labeling library (AAL; Tzourio-Mazoyer et al., 2002), made available in the WFU PickAtlas toolbox (Maldjian et al., 2003), version 2.4, were used to determine the initial set of voxels to be used in the analysis. The masks covered cortical regions, subcortical structures and the cerebellum. BOLD time-series from these voxels were extracted from the functional images (scans) of each participant using MarsBaR (Brett et al., 2002), version 0.42. There were 32,430 voxels in the mask, each one of size of 3 mm × 3 mm × 5 mm. Note that by using such a mask, the number of voxels effectively used to perform the classification was reduced to approximately 34.7% of the original number (93,492). We performed the analysis in two ways; in the first analysis (whole-brain classification), each scan was encoded as an array of size 32,430 containing the values of the voxels that appeared in the comprehensive whole-brain mask; the same mask was used for all participants. In the second analysis, a more directed feature selection (Pereira et al., 2009) was performed, by selecting a subset of voxels based on the masks generated in the previous GLM analysis. In order to assess the effect of the statistical threshold applied to the spatial maps in the classification results, we repeated this procedure for both threshold values (P001 and FWE05). Because these masks were generated individually, the total number of voxels used by the classifiers varied across participants.

The machine learning classifiers were trained using the functional images acquired over the eight runs of Day 1. Sixteen whole-brain scans were acquired each time the Countdown or the PAM task was performed; because each task was performed three times in a run, if the participant correctly responded to all start-task auditory cues, each run would contribute with 48 scans of each task type to the training dataset, totaling 384 scans per task type across the eight runs. When using the contrasts computed in the GLM analysis to select voxels, we used the data from the odd (even) sessions to select the voxels, and the data from the even (odd) sessions to train the classifiers. In that case, the maximum possible number of scans from each task type in the training dataset dropped to half (192). Datasets for the machine learning based analysis were strictly balanced by ensuring that the number of scans in each class was the same; if a participant did not respond to the auditory cue given at the start of a task, e.g., the second block of the Countdown task, all the scans from that block and the scans from the corresponding block of the counterpart task, e.g., the second block of the PAM task in the same run, were excluded from the analysis. This rather severe screening scheme was not used in the GLM analysis, where only the unacknowledged blocks were discarded. The onset times used to determine the scans used to train and test the classifiers were shifted by 4 s (two TRs) in order to account for the hemodynamic delay (Handwerker et al., 2004). Voxel-based data were extracted from the scans of interest using the comprehensive mask or the masks generated from the GLM analysis (P001 and FWE05).

We used linear support-vector machines (SVMs) to train the classifiers (Cortes and Vapnik, 1995), using the implementation in LIBSVM (Chang and Lin, 2011), version 3.11. The parameter *C* (Cortes and Vapnik, 1995), which determines the penalty on misclassified data points, was set to 1. Before testing the classification accuracy on the scans collected on Day 2, we performed a leave-one-run-out cross validation scheme (LOROCV) using the data acquired on Day 1, in order to assess the overall quality of the data: scans from one of the eight runs were put aside (test dataset), while scans from the remaining runs were used to train the classifier (training dataset); the accuracy of the classifier after training was assessed using the test dataset. This procedure was repeated so that data from every run served once as a test dataset; the mean classification accuracy was computed based on the results of all iterations of the LOROCV. Next, we trained a classifier using the entire dataset collected on Day 1, and assessed its performance using the data collected in the three last runs of Day 2 (alternating Countdown-PAM). This was done in order to verify how well the performance of the classifiers generalized to different days. We then used the same classifiers to discriminate the scans in the response periods of each one of the five runs of the Binary Answer task. In all analyses, values in the training dataset were scaled to the interval [-1, 1], using the maximum and minimum of each feature (voxel); the same scaling parameters were applied to normalize the test dataset before classification accuracy was assessed.

Classification accuracy for the LOROCV applied to the data collected on Day 1 was computed by dividing the sum of correctly classified scans accumulated over all iterations of the cross-validation with the total number of valid scans remaining after the screening to balance the datasets. The same formula was employed to verify the accuracy of the classifiers trained with the entire dataset of Day 1 on the data collected from the alternating Countdown-PAM task runs of Day 2. The performance of the classifiers when discriminating the data from the Binary Answer task was assessed in two ways. First, we simply derived the percentage of correctly classified response period scans for each participant, over all five runs; because participants had to provide an answer to each sentence over a period covering 12 scans (24 s), if each one of the six response periods was correctly acknowledged with a button press, there would be 72 scans per run, amounting to 360 scans in total. Statistical significance of the single-scan classification accuracy was examined using a balanced-block permutation test (Schreiber and Krekelberg, 2013), which preserves the blocked structure in the training data imposed by the experimental paradigm when shuﬄing the labels, in order to prevent inflation of significance estimates caused by the temporal correlation across scans resulting from the sluggishness of the hemodynamic response. The second approach was to apply a majority vote on each response period, where the response to a given sentence was computed as being the most frequently occurring output (yes/no) returned by the classifier in the subsequent response period. In order to prevent ties, we performed the majority vote on the first 11 scans (out of 12) of each response period. To examine the effect of the duration of the response period, we also assessed the classification performance using different number of scans (3, 5, 7, 9).

## Results

### Behavioral Results

All participants but one diligently responded to all auditory cues signaling the start of a task block at Day 1 (one participant missed one cue). On Day 2, participants gave responses to all relevant auditory cues, in both the Binary Answer task and the alternating Countdown-PAM task. At debriefing time, participants used a scale of 0: *low* to 10: *high* to rate the *pleasantness* and *vividness* of the memories recollected during the experiment. The average *pleasantness* across participants was 8.06 with standard deviation (SD) 0.90, on Day 1, and 7.61 (SD = 1.36) on Day 2. Meanwhile the average *vividness* reported by the participants was 7.58 (SD = 1.43) on Day 1, and 8.42 (SD = 1.28) on Day 2. All participants declared that they recollected the same memories in the two tasks performed on Day 2.

### MVPA Results

**FIGURE 3:**
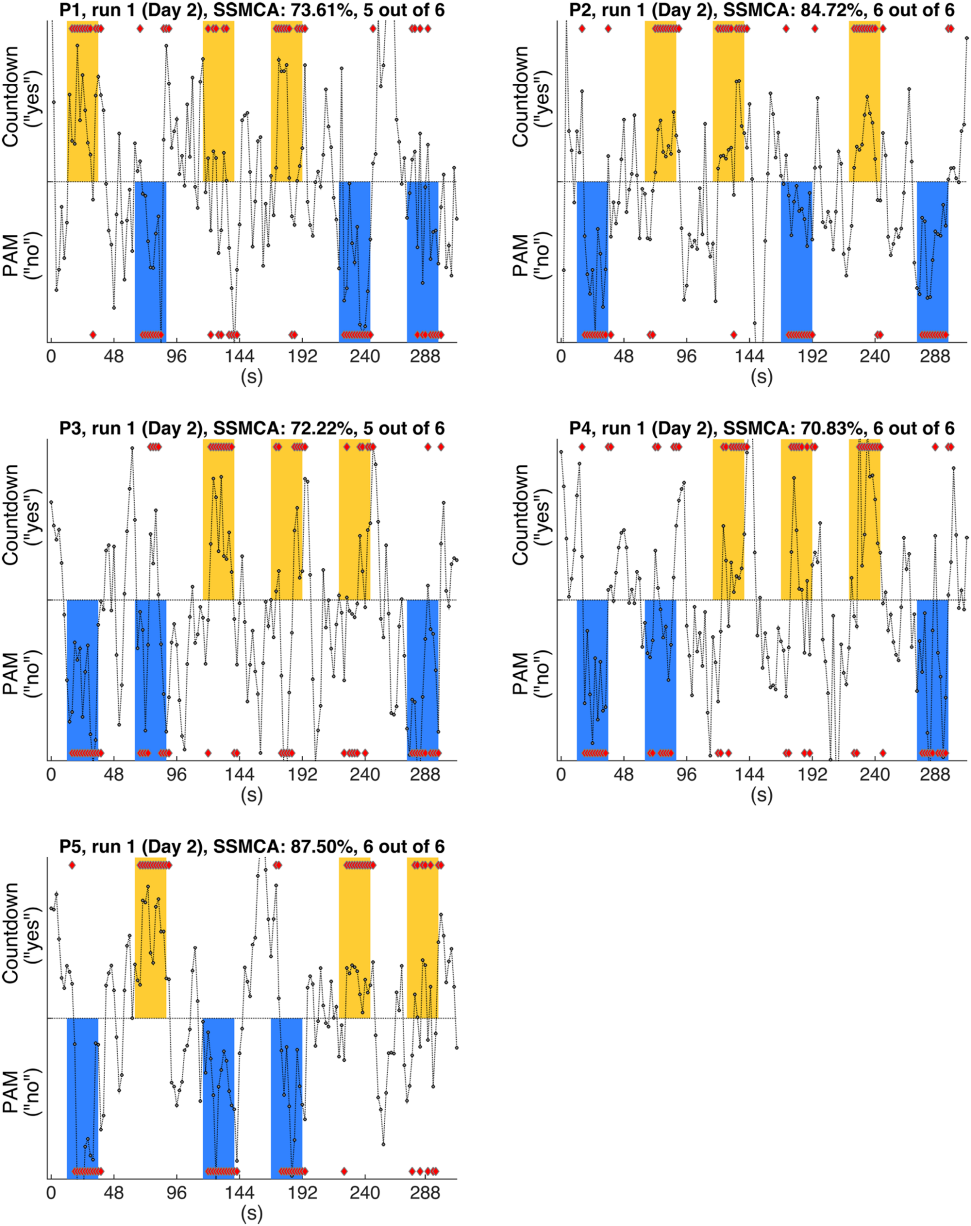
**Gray circles show the timecourses of the actual values output by the individual classifiers when discriminating the whole-brain fMRI scans, for each one of the participants (P1-P5).** Figures show the results of typical runs (run 1, Day 2). By definition, positive outputs indicate that the classifier judged that the fMRI scan belonged to the positive class (Countdown/yes); likewise, negative outputs indicate that the classifier judged that the fMRI scan belonged to the negative class (PAM/no); red diamonds show the assigned class for the scans in the response periods. The yellow and blue bars show the time intervals when participants were supposed to provide answers (yes/no) to each one of the sentences (S1–S6, **Table 1**) by performing the corresponding mental task. Furthermore, yellow bars indicate that the factual answer given by that particular participant to the preceding sentence was ‘yes’, whereas blue bars indicate that the factual answer was ‘no’ (answers were collected via questionnaires at debriefing time). Single scan mean classification accuracy (SSMCA) is the percentage of correctly classified response period scans over the entire run. “*n* out of 6” denotes that *n* sentences out of 6 were correctly classified in that run, when applying the majority vote to the first 11 scans of each response period.

In this analysis, we examined the performance of linear SVM based classifiers in discriminating which mental task a participant was performing based on the fMRI activity patterns contained in a single whole-brain scan. Furthermore, we looked at whether and how this could be used to retrieve binary answers from the participants to a set of six simple sentences. The data used to train the classifiers was collected on Day 1, and classification accuracy was always assessed using data from the same participant (within-participant classification). The results for the whole-brain classification, i.e., using all voxels in the comprehensive AAL mask to train the classifiers, are shown in **Table 2**. We first verified the performance of individual classifiers when tested with scans collected within the same day, by using a LOROCV scheme. Classification accuracies showed to be quite high across all participants (**Table 2**, Column A). Next, we trained a classifier for each one of the participants using the entire dataset collected on Day 1 (alternating Countdown – PAM task, 8 runs), and assessed its performance on the data collected over 3 runs using the same experimental paradigm on Day 2 (**Table 2**, Column B). The mean classification accuracies were overall still high, despite some fluctuations across participants. We did not observe a clear degradation in the results, indicating that classifiers were able to keep comparable performance levels when discriminating scans acquired on a different day. Next, we used the same classifiers to classify data collected when participants performed the Binary Answer task (Day 2, five runs). Though there was a general drop in the mean single-scan classification accuracy levels when compared to the results of the LOROCV, the results were overall still fairly high (**Table 2**, Column C). To test for significance, we conducted a balanced-block permutation test for each one of the participants (5,000 iterations). Results showed that they were significantly above chance at the *p* < 0.003 level. **Figure 3** shows typical time courses of the values output by the classifiers (gray circles), for each one of the participants (first run of the Binary Answer task). Positive values indicate that the classifier decided that the scan in question belonged to the positive class, which was arbitrarily defined to be the Countdown task/yes, whereas negative values indicate that the classifier decided that the scan belonged to the negative class (PAM task/no).

**Table 2:** Mean classification accuracies obtained by the whole-brain linear SVM classifiers trained with data collected on Day 1.

Participant	(A) Day 1 LOROCV (768 scans)	(B) Day 2 Countdown – PAM (288 scans)	(C) Day 2 Binary answer^‡^ (360 scans)	(D) Day 2 Binary answer majority vote
P1	88.7 (5.4)	77.4 (4.8)	73.6 (6.6)	5.0 (1.2) [2]
P2	93.6 (3.9)	92.0 (0.6)	76.9 (5.8)	5.2 (0.8) [3]
P3	87.8^†^ (4.7)	82.3 (4.5)	75.0 (3.3)	5.4 (0.5) [3]
P4	86.1 (6.4)	90.3 (5.1)	75.3 (2.7)	5.8 (0.4) [5]
P5	88.3 (6.8)	95.1 (1.6)	80.8 (10.6)	5.6 (0.9) [4]


*Columns A, B, and C show the mean classification accuracies at the single scan-level, in percent, with the respective standard deviations. The number of tested scans in each one of the cases is shown in the top row in parentheses, unless otherwise stated. The rightmost column (D) shows the mean number of correctly classified sentences in each run, out of the 6, when applying the majority vote in the first 11 scans of each response period; standard deviations are in parentheses. The number of sentences that consistently received the correct factual answer in all five runs of the Binary Answer task is shown in square brackets.*

*^†^Total number of scans was 736.*

*^‡^Results are significant at *p* < 0.003 (balanced-block permutation test)*

We then applied the majority vote in the values output by each classifier during the response periods that followed each one of the sentences. The mean number of correctly classified statements per run for each participant is shown in **Table 2**, Column D, together with the respective standard deviations. All participants had on average more than five out of six statements classified correctly in each run when using the majority vote. Because the same six sentences were used in all five runs, we examined for each participant the number of sentences that were consistently assigned the correct factual response across all runs. That was in the range of 2 to 5 (**Table 2**, Column D, square brackets).

#### Effects of the GLM-Based Feature-Selection

Results in **Table 2** were obtained by training the linear SVMs with examples containing 32,430 voxels (features), as determined by the AAL spatial masks (Tzourio-Mazoyer et al., 2002). In order to examine the effects of a more directed feature selection in the results of the classification, we next used the individual spatial masks generated by the contrasts Countdown > PAM and PAM > Countdown to select the features to be used to train the linear SVMs. In order to avoid biasing the results (Kriegeskorte et al., 2009), the contrasts were computed using the data from the odd (or even) runs, and the resulting masks were applied on the data from the even (or odd) runs to generate the data to train the classifiers. As a result, there was in general a reduction by a factor of 2 in the number of examples in the training dataset. To provide a fairer benchmark, we first recalculated the whole-brain classification results (as obtained for **Table 2**, Column D), just that this time we only used data from the odd or even runs to train the classifiers. The overall mean classification accuracy taking into account the results from the odd and even runs are shown in **Table 3**, Column A. We then repeated the same procedure after performing feature selection using the spatial maps obtained when submitting the results from the GLM analysis to a threshold of *p* < 0.001, uncorrected (**Table 3**, Column B), and to a more conservative *p* < 0.05, corrected for multiple comparisons using family wise error (**Table 3**, Column C). For each participant, **Table 3** shows the overall mean classification performance across the five runs, together with the associated standard deviation. The mean number of consistently and correctly classified sentences is shown in square brackets. When performing feature selection using the results from the GLM-based analysis, the number of resulting features varied across participants, and also between the odd and even runs for the same participant. The mean number of features (voxels) used to train the classifiers is shown in **Table 3** under the column “# feats.”. **Supplementary Figure S1** shows the voxels that were selected by the more inclusive P001 mask, for each one of the participants.

**Table 3:** Mean number of correctly classified sentences when using linear SVM classifiers trained using voxels selected by the results of the GLM analysis (Day 1).

Participant	(A) Whole-brain trained on data from four runs		(B) Voxel-selection based on mask P001		(C) Voxel-selection based on mask FWE05	

		**# feats.**		**# feats.**		**# feats.**
P1	5.2 (0.9) [3.0]	32430	5.5 (0.5) [4.0]	9221.5	5.3 (0.5) [4.0]	3310
P2	5.3 (0.6) [2.5]	32430	5.5 (0.7) [3.5]	8981.5	5.7 (0.7) [4.5]	3517
P3	4.7 (0.8) [3.0]	32430	5.0 (0.7) [3.0]	10479	5.3 (0.6) [4.0]	3841.5
P4	5.5 (0.7) [4.5]	32430	5.7 (0.4) [5.5]	7020	5.5 (0.5) [4.5]	1643
P5	5.5 (0.7) [3.5]	32430	5.5 (0.7) [3.5]	15344.5	5.5 (0.7) [3.5]	8558.5


*Column A shows the mean number of correctly classified sentences per run (majority vote, first 11 scans of each response period) when using the odd runs (1, 3, 5, 7) or the even runs (2, 4, 6, 8) of Day 1 to train the classifiers (results were averaged). Standard deviations are in parentheses, and the number of sentences that were consistently assigned the correct answer in all five runs is in square brackets. Column B and C show the same values when the number of features (voxels) used to represent each example (scan) is reduced by employing spatial masks derived from the results of the GLM analysis under different statistical thresholds (P001 and FWE05, respectively). “# feats.” shows the number of features used by each individual classifier.*

No dramatic changes in the average number of correctly classified sentences were observed across the different settings, though compared to the whole-brain classification results equalized for training dataset size, applying a more directed feature selection (P001 and FWE05) seems to slightly improve the results. More remarkably, the benefits of feature selection are clearer when looking at the number of consistently classified sentences; results of three participants improved when applying some sort of feature selection (P001 or FWE05), compared to the whole-brain classification (in the other two participants, the results did no change). Moreover, the magnitude of the improvement appears to be marginally greater when using the more conservative statistical threshold to select the voxels (FWE05).

#### Response Period Duration Effects

**FIGURE 4:**
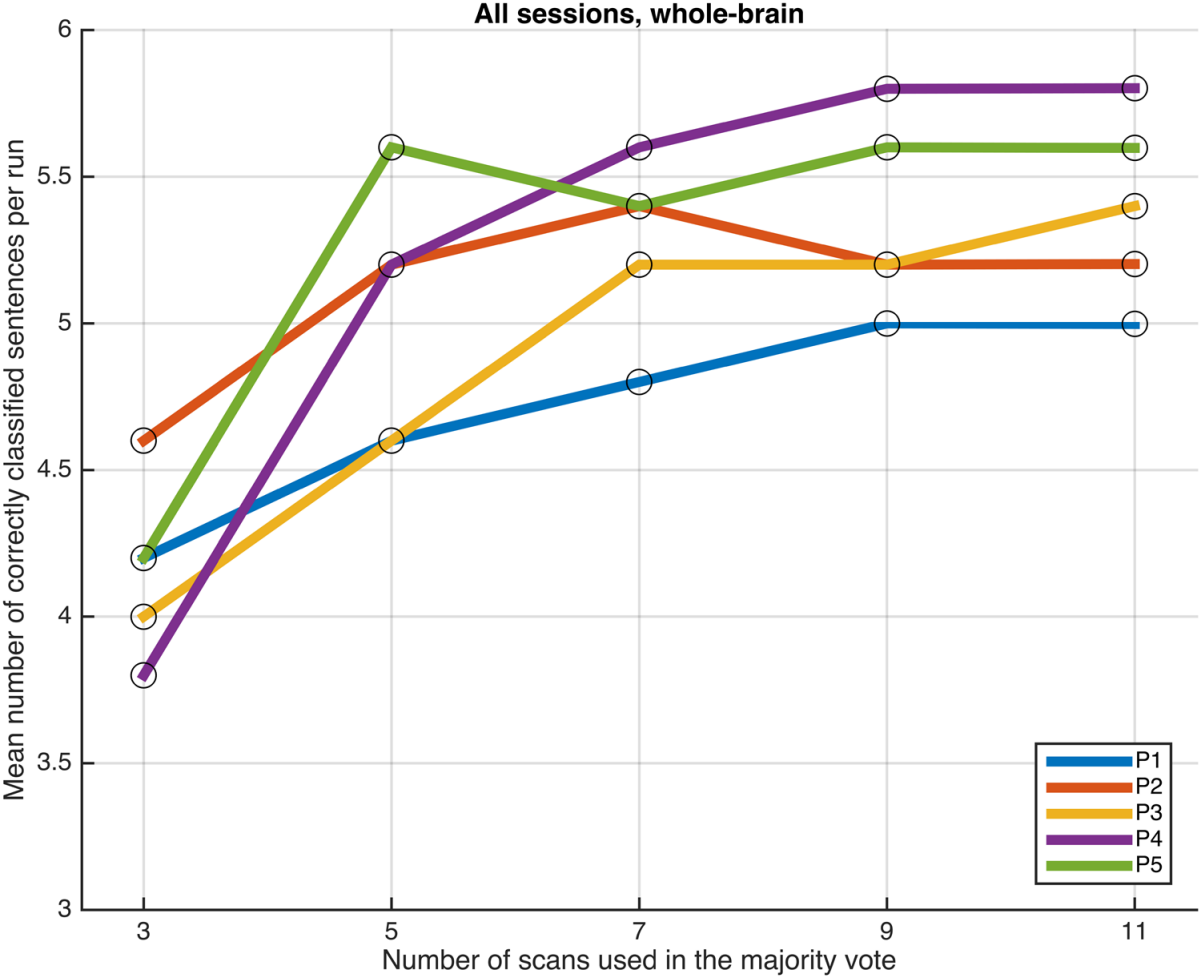
**Progression of the mean number of correctly classified sentences per run as a function of the number of scans used in the majority vote, when training the classifiers on data from eight runs, using the voxels in the whole-brain comprehensive mask**.

**FIGURE 5:**
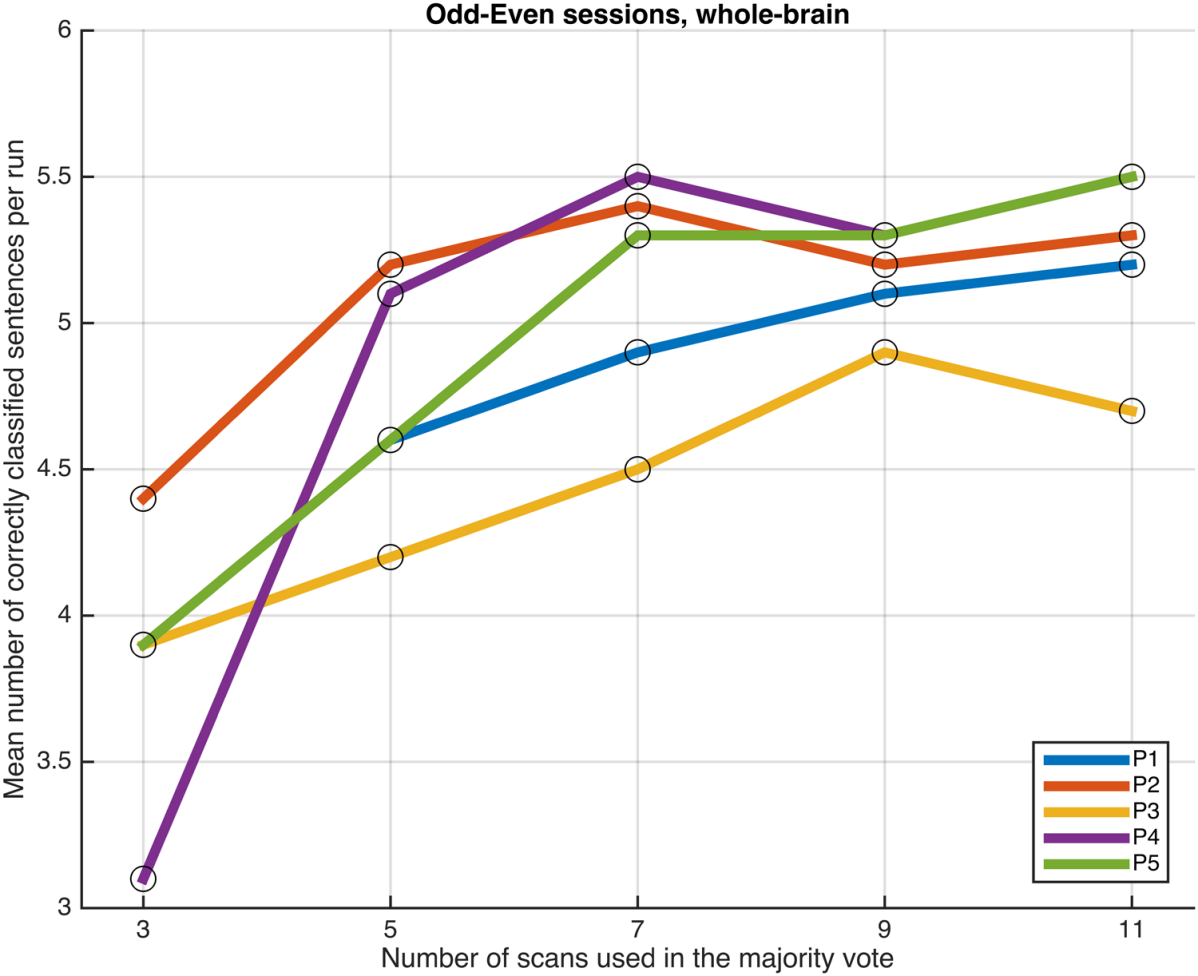
**Progression of the mean number of correctly classified sentences per run as a function of the number of scans used in the majority votes, when training the classifiers on data from four runs, using the voxels in the whole-brain comprehensive mask.** The results from the odd and even runs are averaged.

**FIGURE 6:**
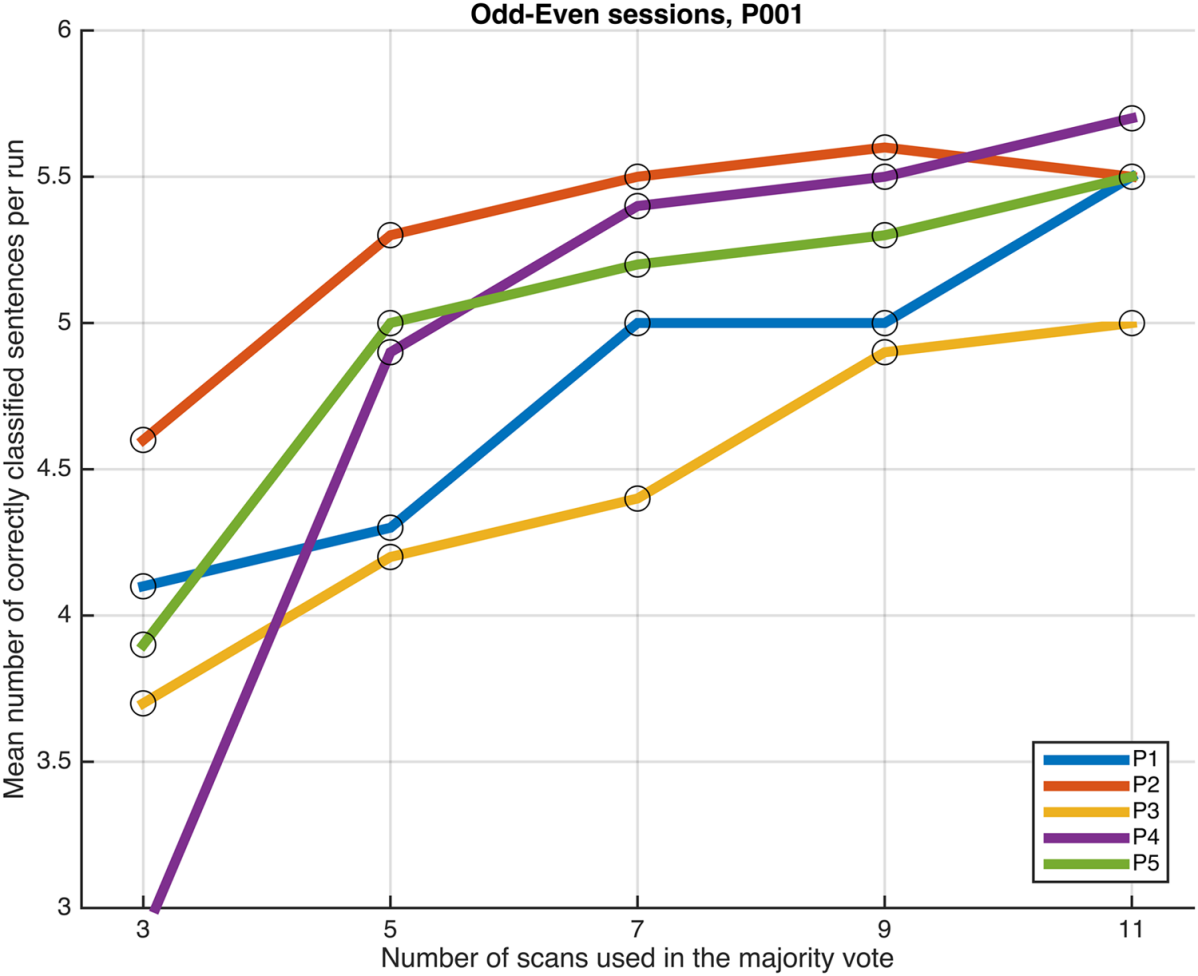
**Progression of the mean number of correctly classified sentences per run as a function of the number of scans used in the majority vote, when training the classifiers on data from four runs, using the voxels in the P001 mask.** The results from the odd and even runs are averaged.

**FIGURE 7:**
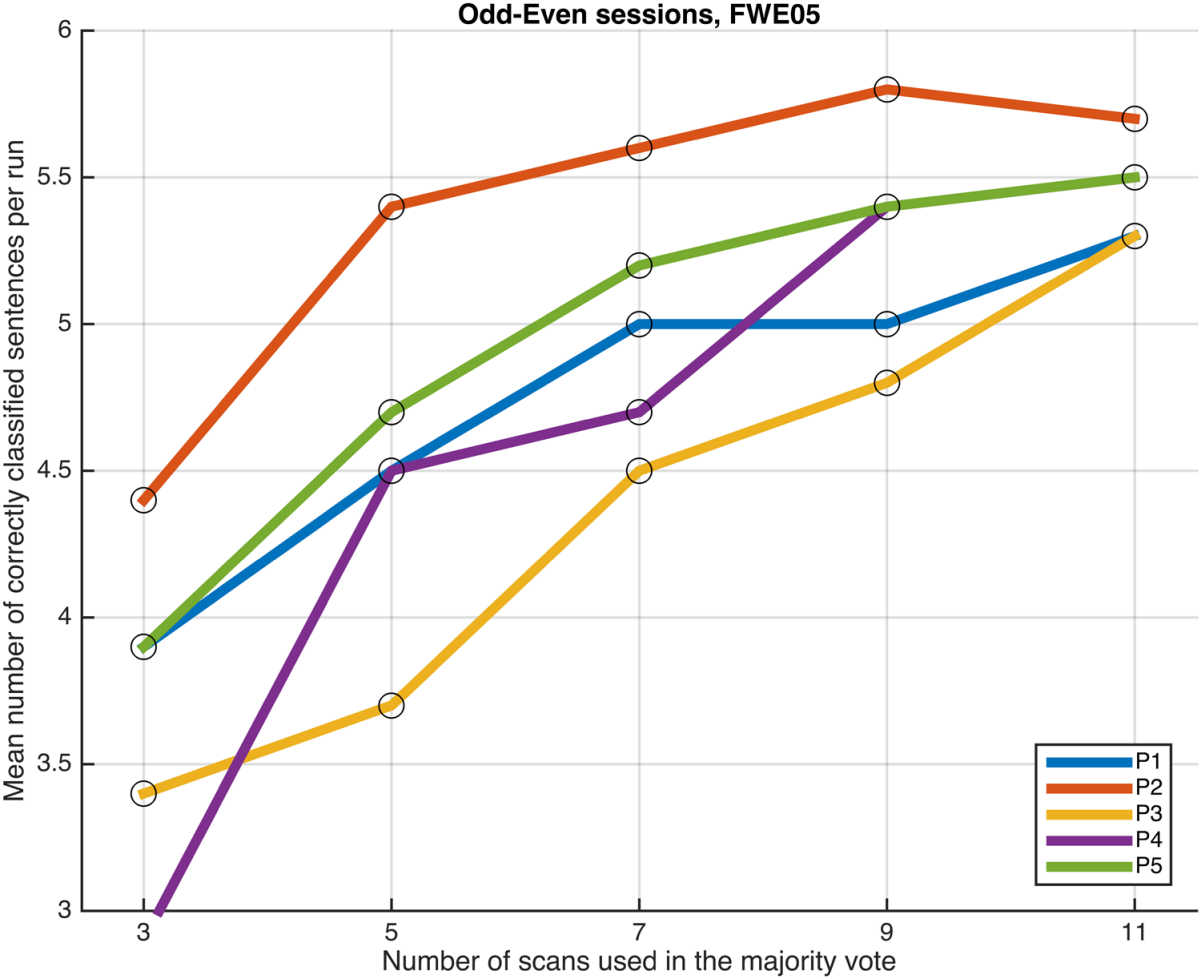
**Progression of the mean number of correctly classified sentences per run as a function of the number of scans used in the majority vote, when training the classifiers on data from four runs, using the voxels in the FWE05 mask.** The results from the odd and even runs are averaged.

Next, we examined whether and how the classification performance was affected when fewer scans were taken into consideration in the majority vote, i.e., in effect, using shorter periods of time when retrieving the answer to a statement. **Figures 4**–**7** show how the mean number of correctly classified sentences per run developed when the first 3, 5, 7, 9, and 11 scans were used in the majority vote, when using the entire dataset of whole-brain scans to train the classifiers (eight runs), half the dataset, half the dataset with the P001 and FWE05 masks, respectively. As a general rule, employing longer periods of time to retrieve answers leads to better performance, across different settings and participants. Among the tested time intervals, the greatest enhancement was achieved when extending the number of tested scans from 3 (6 s) to 5 (10 s). For most participants, and across all settings, performance steadily improved as the number of scans included in the majority vote increased. Furthermore, classification performance only reached a visible plateau when using data from eight runs to train the classifiers (**Figure 4**), suggesting that increasing the length of the response period beyond 22 seconds is not likely to improve performance in that case. Such plateauing was not observed in the other settings (**Figures 5**–**7**), indicating that this is likely to be an effect associated with the size of the training dataset.

## Discussion

Advances in neuroimaging technology, most remarkably, functional magnetic resonance imaging (fMRI), have opened the possibility of using such techniques in bedside clinical applications related to the disorders of consciousness, including attempts to detect covert awareness (Fernández-Espejo and Owen, 2013), establishing simple communication with patients in a vegetative state or minimally conscious state (Monti et al., 2010; Sorger et al., 2012; Naci et al., 2013), and unraveling signs that could enhance the prognosis or guide therapeutic decisions (Di et al., 2008). Among those, devising neuroimaging-based communication systems is of particular importance due to its implications in the overall quality of life and wellbeing of patients in these populations (Owen et al., 2009). In this proof-of-concept study, we examined the feasibility of employing single fMRI scans to retrieve binary answers from healthy participants using MVPA. During the experiment, participants listened to 6 sentences and were instructed to respond by performing for 24 s the Countdown task if that sentence was true (yes) or the PAM task otherwise (no). Linear SVM classifiers were trained for each individual using data collected on a different day. At the single scan level, mean classification accuracies were fairly high, and significantly above chance for all five participants (**Table 2**, Column C).

When applying the majority vote in the first 11 scans of each response period, the mean number of correctly answered sentences per run was 5 or more, out of 6, for all participants (**Table 2**, Column D). This is an encouraging finding given that these results were obtained even without resorting to any feature selection scheme, and especially when considering that any feature selection strategy is likely to effectively reduce the amount of data that can be actually used to train the classifiers. Nevertheless, even when no feature selection was applied, individual classifiers for all participants but one consistently assigned the correct answer to at least three sentences across all five runs. It remains to be verified whether repeatedly asking the same questions in different trials within the same run, as typically done in other studies (Monti et al., 2010), can positively affect classification performance, as well as the reliability of the retrieved answers. It is also important to note that training and test data were collected on different days, therefore, these results attest to the stability of the voxel-level whole-brain activity patterns evoked by the Countdown and PAM tasks in particular, and self-paced mental tasks in general.

Performing feature selection using the results of a GLM analysis seems to improve the performance of the machine learning classifiers. Though differences in the results yielded by applying the P001 and FWE05 masks were not so clear, all other things being equal, performing some type of directed feature selection seems to improve the results; when using the FWE05 mask the lowest mean number of correctly classified sentences per run across participants was 5.3, whereas the same values were 5.0 and 4.7, when using the P001 mask or all the voxels in the brain (equalized for training dataset size), respectively. In addition, the lowest mean number of consistently correctly classified sentences across all runs was 3.5 when using the FWE05 mask, whereas the same values were 3.0 and 2.5, when using the P001 mask or all the voxels in the brain (equalized for training dataset size), respectively. Based on these results, it appears to be that adopting a more conservative statistical threshold (FWE05) when performing feature selection brings greater benefits in terms of classification performance and reliability. Further investigations using a larger sample of individuals will be needed to better substantiate these findings.

As a general tendency when applying the majority vote, the mean number of correctly classified sentences per run decreases if the number of scans employed to determine an answer is reduced (**Figures 4**–**7**). This is an intuitive finding; given that several sources of noise may potentially affect the data in fMRI scans, results are likely to improve as more scans are added to the voting. Indeed, the greatest gain is obtained when increasing the number of scans from 3 to 5, regardless of the mask used to select the features or the size of the training dataset. On the other end of the curves, classification performance only appeared to reach a plateau when training the classifiers with data from eight runs (**Figure 4**); no signs of plateauing were observed when using data from four runs to train the classifiers, regardless of whether and how feature selection was applied (**Figures 5**–**7**). Judging from the present results, intervals between 18 and 22 s seem to be sufficient to retrieve responses from healthy volunteers when using the majority vote, provided there is enough data to train the classifiers. Though it remains to be verified whether the same parameters – in fact, the entire scheme based on the classification of single fMRI scans - can be applied to effectively enable communication with patients with disorders of consciousness, the potential to rapidly and robustly acquire responses from unresponsive patients is perhaps one aspect where machine learning based classification can have the most significant impact in clinical applications. Previous studies have typically relied on detecting region-specific brain activity modulations by comparing spatio-temporal activation patterns obtained via mass-univariate GLM analysis (Monti et al., 2010; Sorger et al., 2012; Naci et al., 2013). Focusing on changes of activation level in specific locations assumes that the functional organization of the brain, at least in the areas involved with the experimental manipulations used to communicate with the patients, is mostly intact after injury. In contrast, the multivariate approach described in our study relies on data-driven machine learning techniques to extract activation patterns that can be distributed over much larger areas, and that may be much less spatially structured than what is expected to be seen when examining univariate region-specific modulations. The advantage of the MVPA approach becomes most apparent when considering that machine learning techniques can be applied in the absence of *a priori* hypotheses; even residual but regular activation patterns encompassing voxels distributed along different areas of the brain may serve the purpose of discriminating between two mental states. The higher sensitivity of the MVPA to detect distributed patterns of activity may play an important role when using fMRI to communicate with patients, especially when considering that most patients fail to show consistent modulations of region-specific brain activity (Monti et al., 2010).

Another important aspect concerning brain-based communication systems is the length of fMRI scanning time needed to obtain a response. More natural conversational communication might only be possible if the time necessary to collect an answer from a patient is substantially reduced from the current levels, where typically several repetitions of intervals lasting tens of seconds are necessary to reliably retrieve a single response. In (Monti et al., 2010) and (Naci et al., 2013) binary answers were collected after several minutes, while the scheme proposed by (Sorger et al., 2012) could in theory attempt to use the 50 s needed to spell a character to encode a yes/no answer (though in the original study, experimenters deciphered letters by not only by looking at the top letter candidates determined by an automatic decoder but by also taking into account the contextual information provided by the question). In this study, participants provided answers in periods of 24 s, effectively halving the time necessary to recover an answer when compared to the scheme proposed by Sorger et al. (2012). Of course, retrieval time must be weighted by the probability of obtaining a response that matches the factual answer. Though performance varied across participants, the best result when using the majority vote was from participant P4 (**Table 2**), from whom the correct answer was consistently retrieved in five out of six questions, across five runs. That amounts to saying that if the most consistent participant (P4) in our sample had taken part in only one run of the Binary Task, the odds of acquiring the correct answer for each one of the six questions would had been 5/6 (83.33%), which happens to be identical to the result obtained by Monti et al. (2010) from the single patient they tested, though in their study each answer required 5 min to be retrieved. No definite conclusions can be drawn yet because of the limited number of participants in this study, nevertheless, applying the majority vote on groups of fMRI scans to obtain responses seems to be a possible alternative to establish basic communication with unresponsive patients, especially when schemes based on the mass-univariate approach do not yield successful results. Future studies must assess how the current results generalize to a larger sample, and examine alternative ways to improve the performance of the machine learning classifiers, such as deploying non-linear kernels to improve classification accuracy.

There are a few important questions that were not addressed by this study. First, all the results are based on individual classifiers, which were trained and tested using data from the same participant. Though several studies have reported successful across-participant classification in the past (e.g., Mourão-Miranda et al., 2005), studies performing single fMRI scan classification relying on mental tasks that do not resort to external stimuli (visual or otherwise) are more rare. In such settings, mean across-participant classification accuracies are likely to drop when compared to within-participant classification accuracies (e.g., Nawa and Ando, 2014). Devising efficient ways to generalize the results of the classification across different individuals is certainly desirable, though there is the more fundamental question of how similar the voxel-level brain activity patterns elicited by mental tasks such as the ones used in this study are across different individuals. If high performance across-participant classification is made possible, it could minimize or even eliminate the need to collect training data to build a classifier for every particular patient, reducing the burden on patients, and saving on often expensive fMRI scanning time.

Another limitation of this study refers to the choice of the mental tasks used to represent ‘yes’ and ‘no’. Studies on fMRI-based communication schemes typically employ imagery tasks as proxies for the responses, and in that respect our study is no different. The basic requirement that guided our choice of mental tasks was that they should be easy to execute, i.e., not require prior training, and if at all possible, resemble activities that are routinely performed by people in the course of their daily lives, as in the case of the PAM task. (Incidentally, we noticed from the debriefing data that the overlap of the memories recalled on Day 1 and Day 2 was only partial; on Day 2, participants tended to concentrate on a subset of the memories recalled on Day 1.) Nevertheless, even though the mental tasks we selected are easy to perform, the rather unnatural way in which they were employed can in itself become an unwarranted source of stress and fatigue to the patients. In that sense, more intuitive ways of expressing responses, such as the one used in (Yang et al., 2014), may be preferable, provided that the overall classification performance and reliability of the results are not compromised.

## Conflict of Interest Statement

The authors declare that the research was conducted in the absence of any commercial or financial relationships that could be construed as a potential conflict of interest.
