# Supplementary material for: Retrieving Binary Answers Using Whole-Brain Activity Pattern Classification
Source: Front Hum Neurosci. 2015 Dec 23;9:689. doi: 10.3389/fnhum.2015.00689 (PMC4688375; doi:10.3389/fnhum.2015.00689)

Supplementary Material

Figure S1: Individual P001 masks used for feature selection for the 5 participants (P1-P5). Areas in yellow show the voxels that survived a threshold of $p < .001$ (uncorrected) in the contrast Countdown > PAM, likewise, areas in blue show the voxels that survived the same threshold in the contrast PAM > Countdown. Left and right panels show the left and right lateral views of each participant's masks, respectively, overlaid on an inflated template brain.

P1

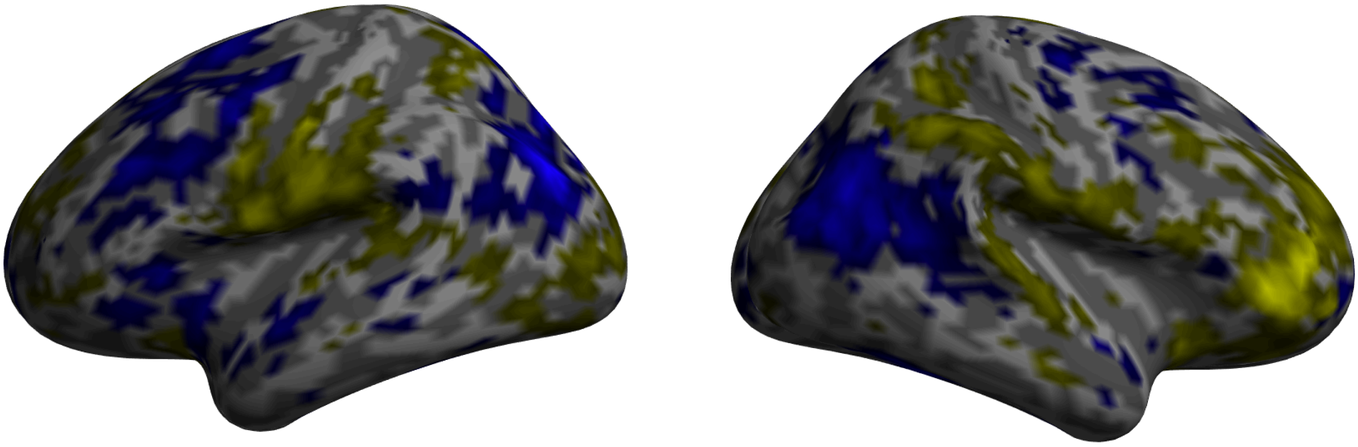

P2

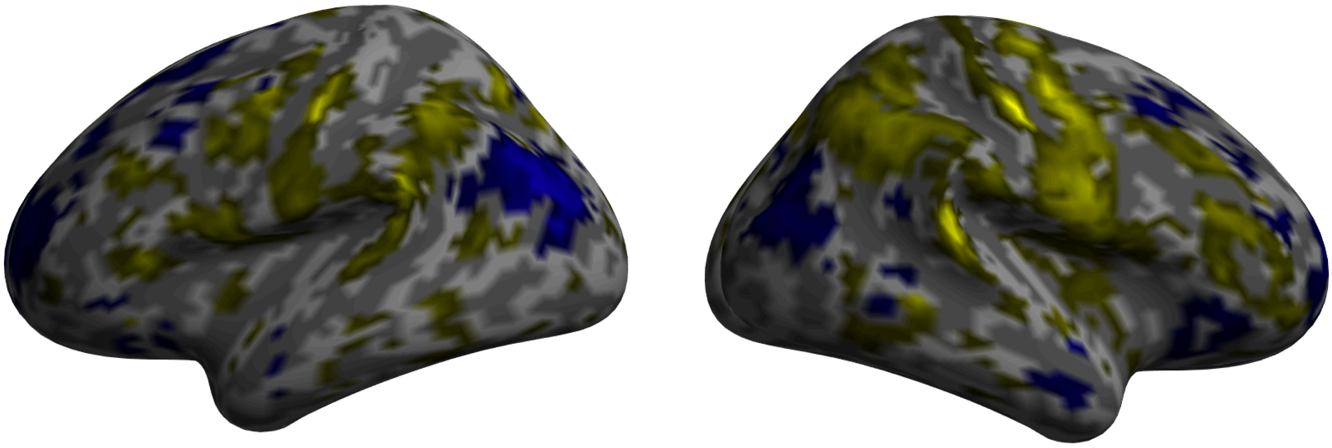

P3

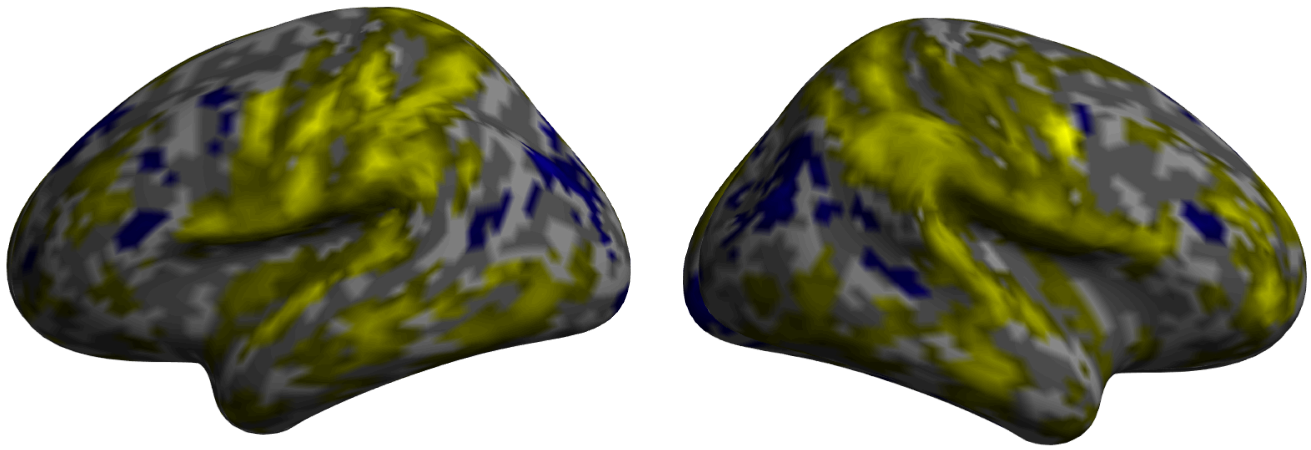

P4

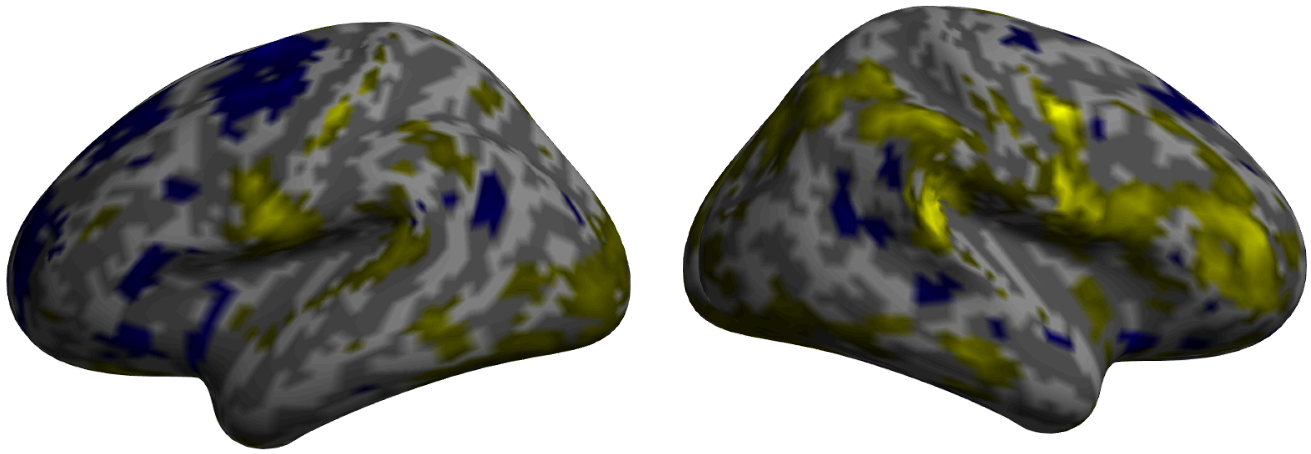

P5

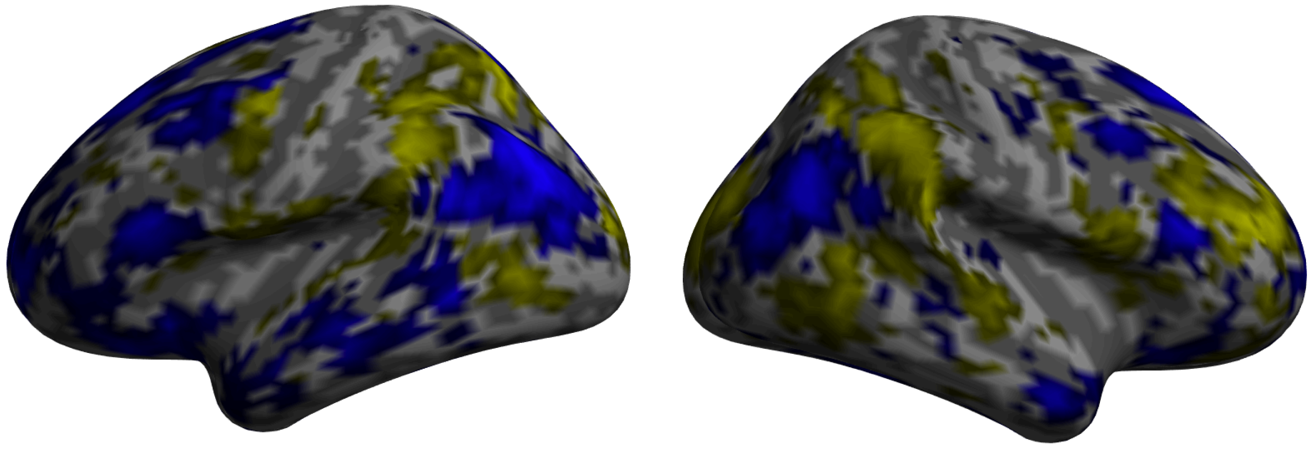

Supplement: Supplementary file 1 [file Image_1.PDF]
